# The Anthropocene condition: evolving through social–ecological transformations

**DOI:** 10.1098/rstb.2022.0255

**Published:** 2024-01-01

**Authors:** Erle C. Ellis

**Affiliations:** ^1^ Department of Geography & Environmental Systems, University of Maryland, Baltimore County, Baltimore, MD 21250, USA; ^2^ Oxford Martin School, University of Oxford, 34 Broad St, Oxford OX1 3BD, UK; ^3^ Leverhulme Centre for Nature Recovery, Environmental Change Institute, School of Geography & Environment, University of Oxford, South Parks Road, Oxford OX1 3QY, UK

**Keywords:** transformative change, visions of sustainability, anthroecology, environmental messaging, human development

## Abstract

Anthropogenic planetary disruptions, from climate change to biodiversity loss, are unprecedented challenges for human societies. Some societies, social groups, cultural practices, technologies and institutions are already disintegrating or disappearing as a result. However, this coupling of socially produced environmental challenges with disruptive social changes—the Anthropocene condition—is not new. From food-producing hunter–gatherers, to farmers, to urban industrial food systems, the current planetary entanglement has its roots in millennia of evolving and accumulating sociocultural capabilities for shaping the cultured environments that our societies have always lived in (sociocultural niche construction). When these transformative capabilities to shape environments are coupled with sociocultural adaptations enabling societies to more effectively shape and live in transformed environments, the social–ecological scales and intensities of these transformations can accelerate through a positive feedback loop of ‘runaway sociocultural niche construction’. Efforts to achieve a better future for both people and planet will depend on guiding this runaway evolutionary process towards better outcomes by redirecting Earth's most disruptive force of nature: the power of human aspirations. To guide this unprecedented planetary force, cultural narratives that appeal to human aspirations for a better future will be more effective than narratives of environmental crisis and overstepping natural boundaries.

This article is part of the theme issue ‘Evolution and sustainability: gathering the strands for an Anthropocene synthesis’.

## 1. Introduction

More than 3 billion years ago, a bacterial species evolved genetic capabilities to harness solar energy. Thus began the most disruptive geological event in Earth's history; the Great Oxidation Event [1]. The oxygen emitted as a byproduct changed Earth forever, killing off species not adapted to oxidizing conditions while enabling the evolution of complex organisms dependent on high energy metabolism, their establishment on land, and an atmosphere enabling fire; all necessary preconditions for Earth's most recent disruptive geological event, the Anthropocene [2–4]. Just as the genetic capabilities of photosynthetic bacteria unlocked entirely new scales of energetic and evolutionary possibility for life on Earth, disrupting everything that came before, so have the unprecedented sociocultural capabilities of human societies and their harnessing of energy beyond the biological world.

Humans, as a species, have long been Earth's ‘ultimate ecosystem engineers' [5, p. 1797]. For millennia, human societies have been transforming Earth's ecological functioning through a broad and accumulating suite of diverse, complex and transformative sociocultural capabilities, from control of fire to nuclear energy, that are beyond those of any prior non-hominin species [6–11]. Global climate change, biodiversity losses and other anthropogenic planetary changes all began long before the industrial age [2,6–11].

The grand challenge for the evolutionary, ecological and sustainability sciences is to understand why and how human societies gained unprecedented capabilities to transform the functioning of an entire planet [7]. Such an understanding may enable the sciences to more effectively address the grand societal challenge of the Anthropocene: to achieve the agency needed to guide these capabilities towards shaping a better future for people and the rest of nature.

The aim here is to outline some basic principles for an evolutionary understanding of why human societies emerged as Earth's newest ‘force of nature’ (sensu [12]) and how this novel force might be guided towards better outcomes for people and planet. The starting point is the recognition that human societies have, for millennia, been evolving transformative solutions to novel social and environmental challenges of their own making [7]. From social norms, irrigation systems and granaries, to environmental protection agencies, electrical grids and international climate agreements [13], human societal capabilities to address social–environmental challenges are increasingly shaping the fate of all life on Earth.

Evidence will be presented to support two claims. The first is that societal capabilities already exist to shape a far better planetary future than the one being shaped now. The second is that societal agency to deploy these capabilities depends on widespread recognition that they exist, together with their empowerment through broadly shared aspirational demands for the better future they make possible. Strategies for increasing societal agency through shared aspirational narratives to shape a better future will be discussed in relation to narratives emphasizing natural planetary limits to human sociocultural capabilities.

### (a) Crisis as condition

The Anthropocene is readily understood as a crisis: a time of unprecedented risks to both humanity and the rest of life on Earth [14,15]. There is simply no precedent for a growing population of more than 8 billion people on Earth, which, together with domestic livestock, already compose more than 90% of all mammal biomass (160 Mt; [16,17]); a biomass similar to all social insects combined (approx. 13 000 species; [18]). Agriculture, settlements and infrastructure now cover more than 40% of Earth's land surface, directly or indirectly transforming ecosystems and habitats across more than 75% of Earth's ice-free land area, leading to rapid planetary declines in biodiversity [11,19]. Combusting fossil fuels for energy, clearing forests, tilling soils and other greenhouse-gas-emitting practices are heating Earth faster than any time since the dinosaurs and the planet is already hotter than it is been for at least 100 000 years [20]. These are just a few among the many disruptive social–ecological challenges that define the Anthropocene as a crisis.

The manifold planetary disruptions of the Anthropocene are unprecedented challenges for human societies and for the rest of nature. Yet human societies have always lived in novel social–ecological conditions of their own making [6,7,9]. When faced with unprecedented social–ecological challenges emerging either externally (e.g. prolonged drought, devasting floods, colonial invasion, epidemic disease, pest outbreaks) or internally (e.g. extreme social inequalities, civil war, soil erosion, resource depletion), or through interactions among external and internal challenges, some societies have disintegrated while others have adapted to, learned from these and transformed themselves, thriving on for centuries to millennia [7,21–27].

The lesson from deep history is clear. Human societies have faced unprecedented challenges countless times since prehistory and the results depend more on how societies have addressed them than on the environmental conditions they faced. Some proved incapable of weathering even modest challenges. Others evolved and accumulated sociocultural capabilities to thrive under even harsher conditions. Complex societies have long sustained themselves in some of the most extreme environments on Earth, including hot deserts and polar regions, through large-scale systems of cooperative cultural and material exchange (e.g. sharing expertise, food storage and distribution systems), dynamic subsistence strategies (e.g. diversification of food sources, seasonal mobility, migration), environmental management practices (protective clothing and settlements, hydraulic infrastructures, irrigation) and the adoption of drought and cold resistant crops and livestock [28]. The basis for surviving and thriving in challenging environments—human-made or otherwise—is determined by the sociocultural capabilities of societies to adapt to, learn from and transform themselves to address these challenges [7,21–28].

The first principle for understanding the Anthropocene entanglement of social and planetary change is that this disruptive condition did not result from a lack of human capabilities to adapt to, shape or sustain the novel social–ecological systems (SES) that sustain human societies. Rather, it is only because human societies evolved unprecedented sociocultural capabilities and agency in shaping their social and ecological environments over thousands of years that human populations are now thriving at levels beyond those of any other species in Earth's history [7,26,28–33]. For better and for worse, the Anthropocene condition of disruptive planetary change is coupled with unprecedented sociocultural capabilities to shape the SES that have always sustained human societies [7].

## 2. Anthroecology theory: an evolutionary theory of the Anthropocene

Anthroecology theory is an evolutionary synthesis aimed at understanding why human societies evolved unprecedented capabilities to transform planetary ecology and how the dynamics of these ongoing transformations have unfolded across the planet over millennia [7,34]. The starting point is the observation of unrivalled human capabilities to accumulate culture through social learning and to live cooperatively within societies and environments shaped by culture [7,26,32,35–41]. Anthroecology theory builds on the Extended Evolutionary Synthesis [29,42] to explain the evolutionary processes set in motion by these exceptional capabilities by integrating theories on ecosystem engineering and niche construction (ecological inheritance; [43,44]), cultural evolution (cultural inheritance; [45–48]), human ultrasociality [30,49,50] and multi-level selection [7,34,48,51–53]. Taken together, these theories explain the accumulation and diversification of unprecedented human sociocultural capabilities for constructing the human social- and ecological-niche as an evolutionary process of ‘sociocultural niche construction’ across human generational time. Understanding this long-term evolutionary process is critical to understanding the increasingly planetary entanglement of social–ecological change that defines the Anthropocene condition [7].

Social-Ecological Systems (SES) theory explains the coupled dynamics of human societies and ecosystems through the lens of complex adaptive systems theory [54–57]. In this way, changes in society and ecosystems are understood as the emergent consequences of feedbacks among system components and patterns of system dynamics are described in terms of their relative stability (e.g. resilience, multiple equilibria) and relative change (adaptive cycles, regime shifts, transformations, panarchy) [55]. SES theory includes learning processes, human agency, institutions and governance systems and its core model of resilience has been adapted in efforts to help guide beneficial transformative changes [55,57,58]. Increasingly, SES theory is being applied to understand and address planetary challenges through the rubric of stabilizing the Earth system in Holocene-like conditions [56,59].

Anthroecology theory offers a different approach. While SES theory is based on understanding systems feedbacks and capacities relating to pathways of development, adaptation and transformation, anthroecology theory aims at understanding long-term evolutionary changes in human capabilities to shape societies and ecosystems and their consequences. In other words, anthroecology theory focuses on explaining the ongoing evolution of transformational possibilities. Evolutionary changes in human cultural traits and their transmission and inheritance are central to anthroecology theory because these are the main determinants of human societal capabilities and because cultural traits, including norms, institutions and technologies, can evolve, reproduce and spread much faster than genetic and other traits [7,60]. Cultural traits are, therefore, increasingly favoured by selection processes under increasingly dynamic environmental conditions, including those produced by many human societies [7,60].

Human sociocultural capabilities to engineer ecosystems, from using fire to clear land, to propagating favoured species, to agriculture, to industrial food systems, have evolved and accumulated over millennia, producing both beneficial and harmful ecological inheritances, from increased ecosystem productivity to soil erosion and pollution [7,11,61,62]. Over the same interval, these increasingly complex and intensive niche construction capabilities have tended to appear in parallel with the increasingly rich diversity and complexity of human cultural practices, technologies, institutions, norms, identities and values that structure human social relations, social groups and societies, including increasing dependence on non-kin exchange and other forms of cooperation that together define humans as Earth's first ultrasocial species [7,30,34,49,50].

**Figure 1.**
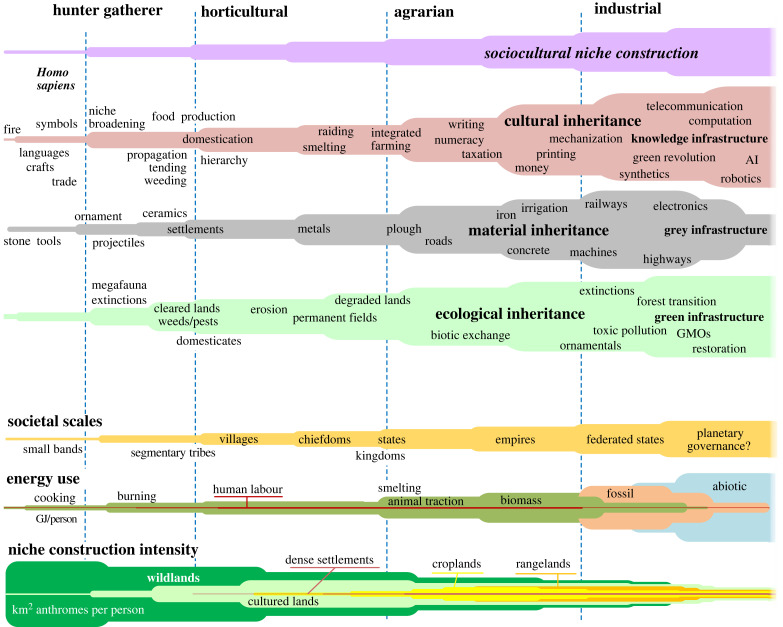
A stylized depiction of long-term evolutionary patterns of transformative anthroecological change, highlighting major regime shifts in sociocultural niche construction, cultural, ecological and material inheritances, societal scales, energy use per capita and niche construction intensity (anthrome area per capita). The linear appearance of this chart is for illustrative purposes only; patterns of change in sociocultural evolution are nonlinear, nondeterministic, and more appropriately depicted as a tree with interconnected branches—as a fabric of coevolution [41]. (GJ, gigajoules; GMO, genetically modified organisms; AI, artificial intelligence.) Based on fig. 3 in [7]. (Online version in colour.)

Like all evolutionary processes, sociocultural evolution is open-ended, diversifying, nondeterministic and generally unpredictable. Most changes in sociocultural niche construction are incremental and gradual, resulting from innovation, selection, accumulation, drift and diversification of sociocultural capabilities within and across societies. Nevertheless, across the tangled web of sociocultural history, some convergent general patterns are observable. With notable exceptions in highly productive and stable coastal and wetland environments, increasingly complex, specialized and larger-scale societies tend to be associated with increasingly intensive, complex and socially coordinated forms of sociocultural niche construction (figure 1; [7,34]). Anthroecology theory explains this long-term trend towards larger-scale societies sustained by increasingly transformative ecosystem engineering as the product of a runaway evolutionary process of sociocultural niche construction [7,34].

### (a) The runaway effect

Runaway cultural niche construction occurs when cultural practices for ecosystem engineering, such as livestock husbandry, enhance selection for cultural or genetic traits that are adaptive to engineered ecosystems, like sedentism or lactose tolerance, such that these in turn select for even greater dependence on cultural niche construction [63,64]. For example, a cultural trait (dairy livestock management) creates a beneficial ecological inheritance (availability of milk) that enhances the fitness of a genetic trait (lactose tolerance), creating a positive evolutionary feedback loop that drives further increases in the reproduction of both traits. This process of runaway selection leads to high frequencies of both traits and an increasing dependence on dairy livestock culture.

Another example is niche broadening, in which cultural traits enabling more intensive use of a broader range of species become increasingly adaptive in response to the depletion of preferred species, which is in turn caused by these more intensive capabilities for hunting, fishing and foraging [65]. In this case, cultural traits enabling more intensive use of underused species generates a negative ecological inheritance (lower availability of preferred species) that enhances selection for cultural traits enabling even more intensive use of an even broader range of species. The resulting positive feedback loop leads to societies increasingly dependent on and capable of more intensively using an ever-broader range of species and an increasingly dynamic ecological niche shaped by these cultural capabilities.

Runaway sociocultural niche construction goes one step further than runaway cultural niche construction by including processes of multi-level selection acting on cultural traits beyond the individual level. This enables an evolutionary understanding of selection acting on social groups and societies, and on cultural traits facilitating the emergence and functioning of larger-scale societies defined by increasingly diverse and specialized social roles and social groups, larger and denser populations, and larger scales of social cooperation including social exchanges of food and resources [7,34,48,51].

For example, cultural practices for propagating a favoured wild species in a burned and tilled clearing might increase food production—enhancing niche construction intensity (production per unit of land or other resource). Successfully engaging in this practice might then select for groups specialized in food production, which tend towards more sedentary lifeways, differentiating them from more mobile non-producing groups and ultimately shaping increasingly dense, sedentary and food production-dependent hunter–gatherer societies and landscapes [7,11,66]. Surpluses produced by this runaway pathway might also enable the differentiation and expansion of groups not engaged in food procurement or production—increasing the effective scale and social complexity of societies. Over the long term, agrarian societies and domesticated species emerged among food-producing hunter–gatherers through an analogous pathway of runaway coevolution with the wild species they depended on [11,66].

The general trajectory of ever larger-scale societies sustained by increasingly intensive niche construction practices can be explained by runaway sociocultural niche construction (figure 1). Increasing demands by social groups that do not produce food, such as religious elites, nobility, warriors and craftspeople, can select for intensive food-producing groups and cultural practices capable of generating extractable or exchangeable surplus and for social groups specialized in extraction and exchange. At the same time, increases in food and resource supplies further drive growth in elite and other non-producing groups. Together, these selective feedbacks can drive the runaway coevolution of intensive niche construction and larger-scale societies, together with increasing per capita consumption and larger and denser urban populations far from sites of food production.

Runaway sociocultural niche construction is neither inevitable nor inexorably driven in one direction or another. Intensive cereal-based agriculture has been associated with increasingly larger-scale, hierarchical, unequal and oppressive societies [67]. Yet archaeological evidence also confirms that cereal producing societies evolved many different forms, including many without high levels of hierarchical social differentiation [68,69]. Fishing, foraging and hunting also sustained the evolution of deeply unequal larger-scale societies, especially in productive coastal and wetland regions [68,69]. Nevertheless, archaeological and historical evidence confirms that agricultural intensity and societal scale and hierarchy often increase together over time. This convergent pathway of runaway sociocultural niche construction is also the general pathway leading to the planetary transformations of the Anthropocene.

Positive feedbacks between societal scale and niche construction intensity are the cause of Earth's accelerating planetary transformation [7]. Rapidly urbanizing societies dependent on extensive exchange networks select for increasingly intensive, technologically complex and productive food systems using high external inputs of energy, nutrients and other resources [7,11,70]. Now, most people live in increasingly globalized and urbanized societies sustained by increasingly intensive industrial food systems interconnected through commodity chains stretching across the planet. The planetary disruptions caused by these increasingly affluent large-scale industrial societies, from climate change and pollution to biodiversity losses, are what now define the Anthropocene as a crisis [7,71].

## 3. Guiding social-ecological transformations

Current rates and scales of anthropogenic global change have been described as a ‘planetary emergency’ driving an irreversible regime shift to a less habitable, ‘hothouse’ climate state [72]. Whether this planetary tipping point has already been passed or might still be avoided will most likely remain unknowable for decades to centuries ([73–76, p. 202], [77, p. 980]). In the words of Hans-Joachim Schellnhuber:

The future of the Earth System cannot be predicted—due to irreducible cognitive and voluntative uncertainties. [78, p. 116]

There is nothing desirable about the hotter, more polluted and less biodiverse planet now being shaped by industrial societies. However, the critical tipping point of the Anthropocene was passed long ago. Just as photosynthesis unleashed entirely new and disruptive evolutionary trajectories for all life on Earth, the transformative sociocultural capabilities of human societies are unleashing unprecedented planetary possibilities, for better and for worse. Either way, the biogeophysical capacities of this planet to sustain people and the rest of life depend at least as much on human sociocultural capabilities as on the self-regulatory feedbacks of the Earth system. The question of whether human societies will shape a better Anthropocene than the one they are shaping now cannot be answered by geophysics, Earth system science or even technological innovation.

### (a) Sociocultural capabilities shape the planet

The ‘natural’ planetary conditions of the Holocene are not, and have never been, the ‘safe operating space’ for human societies (*sensu* [79]). There is nothing unprecedented about extreme droughts, floods, epidemics, pest invasions, colonial exploitation, social unrest and civil war [21,27,68,80]. The ecosystems and food systems that sustain people, including Earth's least and most productive and biodiverse landscapes and seascapes, have long been shaped and sustained by sociocultural niche construction [7,11,66,81–86]. Even the remarkably stable interglacial climate of the past 10 000 years would already be cooling back to a glacial state without past emissions of greenhouse gases from early agriculture, or at least without industrial emissions [87–89]. A past planetary condition of stable and balanced climates, ecosystems, societies and social–ecological interactions is based more on wishful thinking than on evidence [78,90,91]. The same holds for Earth's future.

Earth is not capable of sustaining the vast majority of existing and future human populations without increasingly intensive sociocultural practices for use of land, biota, water, energy and other resources, together with unprecedented scales of social cooperation in sharing them [7,92]. To the extent that safe operating spaces do exist for human societies, these are shaped and sustained by the sociocultural capabilities that these societies have evolved and accumulated. A brief list might begin with protective clothing, stone tools and fire, and lead on to niche broadening, the propagation of favoured species, institutions for sharing and exchanging resources across groups, agriculture, irrigation, public granaries, community resource management, educational systems, global supply chains, healthcare, social security systems, electrical grids, air conditioning, environmental protection agencies, international agreements, real-time global communications and clean energy.

### (b) Capabilities are not enough

The planetary conditions shaped by the Anthropocene entanglement are increasingly unstable, unpredictable and inextricably coupled with the dynamics of human societies [7,93,94]. Yet the unprecedented planetary challenges of global climate change, biodiversity loss and so many others are not caused by a lack of human sociocultural capabilities to address them. Rather, these negative planetary conditions represent failures to address these challenges by the social groups and societies directly responsible for them.

Sociocultural capabilities define the possibilities of what societies can do. However, capabilities are not enough. Even when societies are faced with existential environmental and social challenges, the transformative societal capabilities to address them, including effective forms of governance, sustainable intensive agriculture and solar power, can languish for generations without effective investments, improvements and widespread implementation [95].

At larger societal scales, agency to deploy transformative sociocultural capabilities is enabled only through high levels of sustained cooperation among social groups—a level of cooperation achievable only when societies are capable of delivering what is perceived, based on their cultures, as a fair sharing of societal benefits and burdens in achieving the common good [7,13,21,22,25–27,32,53,96–100]. Societies that are able to deliver on their collective aspirations for the common good, especially in the face of unprecedented social or environmental challenges, tend to endure and to thrive, while societies unable to deliver these basic social goods tend to experience conflict and disintegration. Indeed, challenging conditions can even select for higher levels of collective aspiration and action, social cohesion among groups and greater societal agency in achieving societal aspirations for a better future.

The essential question of the Anthropocene is whether the most powerful, affluent, technologically capable and interconnected social groups and societies that have ever existed on Earth will deliver on their shared aspirations for a better future for people and planet.

### (c) Evolving through disruption

The history of sociocultural evolution is not that of a unified humanity progressing towards a common destiny. Rather, the Anthropocene entanglement represents an evolving interplay of increasingly diverse, differentiated and interacting societies, social groups, people, non-human organisms and environments.

Across this heterogenous and dynamic planetary history, there are probably as many examples of societal regime shifts from larger, denser, more complex and more ecologically transformative societies to smaller, more dispersed, less complex and less intensive societies as the reverse [21,67,68,91,101]. Some, if not most of these societal downscalings and disintegrations are associated with social and/or environmental challenges, internal and/or external. Yet many have also been aspirational, beneficial and shaped intentionally, at least by some social groups within these societies, through desertions, outmigration, revolts and other acts of resistance against unacceptable social or ecological conditions [21,67,68,80,101]. The same can be said of some societal upscalings from smaller- to larger-scale societies. Other societies have remained remarkably stable in societal scale, sociocultural conditions and niche construction intensities even in the face of unprecedented challenges that overwhelmed the adaptive capacities of others.

The planetary scale disruptions of the Anthropocene have largely been produced through the runaway upscaling of recent colonial and industrial societies [7,71]. Nevertheless, all three sociocultural niche construction pathways—upscaling, downscaling and stabilizing—have operated for most of human history and remain active today, and all three offer opportunities to shape a better planetary future for people and the rest of nature.

### (d) Disruptive upscaling: intensifying, energizing and densifying

Major transformative increases in the scale and intensity of sociocultural niche construction, from hunter–gatherer food production to low-intensity agriculture to high intensity industrial production are generally linked with substantial increases in capacities to use non-human energy to power societal functions, including niche construction (figure 1). Just as photosynthesis expanded the energetic scale of the entire biosphere, energy inputs from biomass burning (cooking, heating, clearing land for favoured species), domesticated livestock, hydropower, fossil fuels, solar, wind and nuclear energy have expanded the scope and scale of what is possible for human societies and the anthropogenic biosphere. Increasing energy use and niche construction intensity are also associated with increasingly hierarchical larger-scale societies [92,102].

There is no one pattern of societal upscaling. Nevertheless, larger-scale societies generally include larger, more affluent populations with more diverse livelihood strategies concentrated in dense settlements sustained by exchange systems dependent on increasingly intensive, productive and efficient use of land, energy and other resources per capita [7,34,102]. Aspirations to access this affluence and other opportunities available in the densest settlements of large-scale societies have long attracted people from less dense regions within and outside their domains [103]. Today, increasingly wealthy and globalized societies are associated with high levels of urbanization, inmigration, wealth, inequality, diversity of social groups, longer healthier lives, increased educational and employment opportunities and other indicators of both human wellbeing and potential for social conflict [70,103–105].

Societal upscaling tends to generate a wide array of unprecedented social–ecological challenges, including extreme social inequality, conflict, resource depletion, environmental pollution (including the vast majority of carbon emissions driving rapid climate change) together with other forms of environmental degradation and biodiversity losses [7,15,59,71,92]. These may at least partly be caused by a tendency towards increasing physical and cultural separation of people and social groups from direct interactions with each other and with the non-human world in larger-scale societies [102,106,107]. Larger-scale societies have also tended to appropriate and colonize the peoples, lands and waters of Earth's most productive regions, displacing, disrupting and damaging the smaller scale societies who long shaped and sustained the productive and biodiverse anthroecosystems of these regions [68,85,108].

### (e) Disruptive downscaling

There are countless examples of societal downscalings around the world, some temporary and others long-term or cyclical, from the Mesopotamians to the Egyptians, Greeks, Maya, Cahokians, Khmer, Romans and Byzantines [21,27,68,80]. Societal downscaling is often portrayed as the inevitable result of overwhelming environmental and social challenges—i.e. as ‘collapse’. However, societal downscalings can also be active, intentional and aspirational. For example, the aspirational struggles of producers resisting exploitation by wealthy elites can lead to the disintegration and reorganization of highly hierarchical and unequal societies, downscaling their exchange relationships, levelling social inequality, dispersing populations beyond urban centres and generally de-intensifying their niche construction practices [21,27,67,68,80,91,101].

The consequences of disruptive downscalings have generally been viewed as negative—as ‘dark ages’—by many historians. Yet these usually involve removing, reorganizing or breaking down oppressive elite groups. Archaeologists increasingly recognize these as the product of aspirational efforts by societal majorities to improve their wellbeing by resisting exploitation through practices ranging from non-compliance to outmigration to revolution [21,27,68,80]. Many disruptive downscalings are also slow enough—across a human generation or longer—to go unnoticed by the majority of their populations [21,27,68,80].

Episodes of societal downscaling often correspond to external environmental or social challenges such as droughts, disease or colonial invasions, especially when sociocultural capabilities to weather or combat these challenges are lacking. Nevertheless, societal downscaling in the face of challenges is usually neither inevitable nor unstoppable. A wide array of societal responses to such challenges can mitigate their effects, including adaptations and social changes that increase capacities to cooperate and share resources, such as the reduction of grain taxes, construction of communal granaries, irrigation systems and other institutions and infrastructures that produce the shared social benefits that inspire sustained participation in larger-scale societies [21,23–25,27,32,97].

### (f) Stabilizing selection

When cultural traits enable societies to adapt to environmental or social challenges without transformative changes in societal scales either up or down, this represents a process of stabilizing selection. For example, cultural institutions may limit hunting and foraging pressures on species in decline, elites may redistribute resources, wealth or power and cultural institutions, including costly punishments and shared aspirational narratives may enhance incentives for cooperation and limit overexploitation, as with traditional kinship beliefs connecting people, social groups, species and spaces [28,109].

Many hunter–gatherer, horticultural and agrarian societies have sustained themselves for centuries to millennia without causing disruptive ecological changes [11,28,85,108]. Indeed, many of the most biodiverse, productive and ecologically vibrant regions that remain on Earth persist in areas where Indigenous peoples and traditional lifeways still shape and sustain the anthroecosystems they have always lived in [85,108,110–112].

From hunter–gatherers to farmers to urbanites, much of what holds societies together, from languages to religions to beliefs about nature and the benefits of cooperation have probably accumulated largely because of stabilizing selection; and over time, the increasing cultural capabilities of human societies to function effectively at different societal scales, from dozens to billions, and to scale up and down, might be considered a kind of social superpower in itself [7,36,50,100].

## 4. Evolving towards a better Anthropocene

The future shaped by the Anthropocene entanglement of social and planetary change is no more predictable than the course of biological evolution after photosynthesis. Indeed, the scales of energy now potentially accessible to human societies, including millions of years of fossilized biomass, solar, wind, hydro, geothermal and nuclear energy, go far beyond the annual energy capacity of the photosynthesis that powered all life on Earth for more than 2 billion years. At the same time, human societies have unlocked unprecedented sociocultural capabilities for real-time global social learning.

What will happen as this planet's most social species accumulates unprecedented social superpowers while unleashing Earth's most disruptive energy transition? The possibilities might seem unlimited, from dystopic catastrophe to interplanetary utopia. However, it would be unwise to bet on either extreme [92]. As with the emergence of photosynthesis, the Anthropocene event will probably continue to shape the evolution of disruptive new possibilities for life on Earth. Like Earth's first cyanobacteria, many human societies will not survive this disruption—and many have already been lost, destroyed or colonized by others. Yet, just as photosynthesis continues to power the evolution of unprecedented biological capabilities, so may the energy unleashed by the unprecedented sociocultural capabilities of human societies.

The Anthropocene event, like the Great Oxidation Event and other disruptive geological events, includes species extinctions followed by intervals of diversifying and stabilizing selection [86]. Likewise, runaway sociocultural niche construction powered by novel energy resources is reorganizing the patterns of life across the planet. The evolution of transformative sociocultural capabilities continues to unlock new possibilities from previously unusable resources, to improve resource-use efficiencies (e.g. land use intensification and other forms of decoupling) and to achieve new economies of scale through urbanization, global exchange networks and other unprecedented social capabilities. This disruptive mix of extinctions, diversification, societal upscaling and increasingly efficient land and resource use powered by increasing use of novel energy resources is not an iron law. Yet it would be equally unwise to expect major departures from this evolutionary trend without major efforts to alter this trajectory.

### (a) Engaging in disruption

The question is not whether the Anthropocene's planetary disruptions will continue, but rather, whether the broader evolutionary dynamics now shaping planetary social–ecological change, including upscaling, downscaling and stabilizing, might be guided towards shaping a better future for people and the rest of nature over the long term. Any such effort must begin with humility. There is and will be no certainty or control over the rapidly evolving planetary social–ecological entanglements of the Anthropocene.

‘Holocene-like’ planetary conditions offer no safe space for people or the rest of nature when exploitive elites dominate human societies. Even the best intended interventions can make matters worse. There is a long history of solving one problem by creating a larger one [98,113], like the use of fossil fuels to overcome the limits of biomass. Efforts to limit or control transformative social–ecological change might only serve the same elite social groups that now benefit the most by maintaining existing social inequalities and systems of extraction, pollution, climate change and biodiversity loss [25,114,115].

Disruption is good when the situation is bad [116]. Efforts to shape a better Anthropocene will need to engage with all modes of sociocultural evolution, including some of the same disruptive upscaling processes that have enabled some human societies to reshape this planet for the worse. Harnessing new forms of energy is disruptive, but climbing the energy ladder from biomass to fossil fuels and potentially to abiotic forms of energy has also clearly benefitted billions of people, while also helping forests and other ecosystems to recover [92,117,118]. Rapid societal upscaling through urbanization and industrial development has disrupted climate and caused biodiversity losses, but has also dramatically improved human wellbeing, and many of the societies that have upscaled the longest and the most are now trending towards stabilizing populations (demographic transitions), ecosystem recoveries (forest transitions) and institutional environmental protections [13,70,104,119], though trends in social inequalities remain troubling [120]. More developed industrial societies have also exhibited some trends towards remediating social inequalities, injustices and related environmental harm [13,104,114,120,121].

The increasingly planetary entanglement of social and ecological change is accelerating confrontations among societies and social groups, exposing unacceptable inequalities and injustices around the world at a time when human social learning is increasingly real-time and global [122]. Challenges to existing elites are ongoing. Cultural values, aspirations and capabilities continue to accumulate—including those for sharing power, opportunities and resources more fairly among people, while also sharing space with the rest of life on Earth. However, transformative changes for the better have only ever come through struggle.

Disruptive upscaling can create novel opportunities for improved human wellbeing while sparing nature. Disruptive downscaling can reduce social inequalities by redistributing power, wealth, land, and other resources. However, there is nothing inevitable about either path. Stabilizing selection might simply maintain current societal structures and dynamics even in the face of their increasingly negative societal and planetary consequences.

### (b) Anthropocene opportunities: human aspirations shape planetary capabilities

The unprecedented entanglements, complexities, uncertainties and challenges of the Anthropocene are matched by unprecedented sociocultural capabilities to shape the human sociocultural niche. Where human aspirations have prioritized better outcomes, some of the same transformative sociocultural processes that are now shaping this planet for the worse are also shaping it for the better [13,70,104,105,121,123]. Imagine, for example, the state of the planet today without high-yielding agricultural technologies, environmental protection agencies or international agreements to protect the ozone layer and endangered species. None would be possible without larger scales of human societal cooperation.

Cautionary tales and crisis narratives have always played important roles in shaping social action, especially when based on powerful shared experiences, like extreme weather events. However, crisis narratives based on abstract and differentially experienced concerns, like the exceedance of a specific global temperature, tipping point or other planetary indicator, especially when attached to actions one cannot personally enact, impossible time limits (e.g. ‘only 12 years to act’) and government failures to act, can cause more anxiety than action [22,124–129]. Even worse, crisis narratives, when developed and deployed primarily by elites, well-meaning or otherwise, can drive divisions, disempowerment and conflict [125,130–132].

Portraying the Anthropocene as an environmental crisis ignores its most important message. When people work together, they can indeed change the world for the better.

Human aspirations are a force of nature in the Anthropocene, and just as there is no single human identity, but many, such are the diverse and evolving cultural aspirations of people, social groups and societies. The aspirations of the few and the powerful can and often do conflict with the needs of the many, as when calls for sustainability conflict with demands for transformative change [25,105,116,133]. Indeed, the archaeological record is rich with the remains of extinct elites who failed to meet such demands. Still, some aspirations may be nearly universal, including health and longevity, desirable lifeways and fair access to social, cultural and ecological opportunities. To achieve planetary scales of societal agency, these and other widely shared aspirational demands will play a central role.

Storytelling and other forms of narrative sharing have always been critical to shaping collective aspirations and actions [134,135]. For example, narratives of relationships between land and people have shaped both settler colonialism and traditional cultural identities. Settler colonial narratives often shaped disastrous outcomes for people and nature. However, for many if not most Indigenous and traditional peoples, cultural narratives of kinship relations and prosocial cooperation are extended to embrace and sustain all living beings and even Earth itself [109,132,136]. Such narratives of kinship and reciprocity exemplify the co-beneficial possibilities of social–ecological evolution in the Anthropocene, underpinning the biodiverse and productive cultural ecosystems long sustained by Indigenous and traditional societies.

The urgency of societal transformations to abate carbon emissions, reverse biodiversity losses and meet other urgent social needs does not mean that narratives of crisis, limits and collapse will more effectively bring people together to shape a better future. To harness the social superpowers of human aspirations, the long-term successes of extended kinship relationships should be seen as foundational.

In increasingly globalized societies, the aspirational narratives of human development, including human rights and fair access to health, education and decent standards of living, have shaped decades of improvement in human wellbeing for billions of people [105,137]. Building on these narratives to include human rights to a ‘clean and healthy environment’ [138], together with narratives calling for rights of nature, multi-species justice and other hopeful aspirational narratives connecting human wellbeing with the wellbeing of the rest of nature are increasingly essential to shape a better planetary future [109,124,127,128,136,139–148]. This is even more critical on a planet where people are increasingly connected with each other electronically, while living lives more physically distanced from daily contact with each other and the rest of life on Earth [106,149].

As with extended kinship, aspirational narratives that deeply connect people with each other and the rest of life will be critical to restore and sustain the mutually beneficial evolutionary feedbacks needed to sustain both. Re-emphasizing the kinship relationships among all living beings—our common evolutionary ancestry—is a start, combined with new ways to connect people and nature, from remote sensing to webcams, to nature apps, to parks and ecotourism. Aspirations for a better future must also make peace with the past through restoration of Indigenous and traditional sovereignty over lands and waters.

The sociocultural capabilities to shape a far better future for people and nature already exist. Human aspirations will continue to drive these capabilities towards shaping the social–ecological conditions for human thriving—the ‘planetary capabilities' that must now sustain both human societies and the rest of life on Earth. The time has come to put the focus where it belongs—on our shared aspirations for a better future and making sure that those in power are accountable to meet these aspirational demands. The way people treat each other shapes the way people treat the rest of nature, and the other way around.

## Data Availability

This article has no additional data.
